# Contemporary flowable bulk-fill resin-based composites: a systematic review

**DOI:** 10.1080/26415275.2023.2175685

**Published:** 2023-02-22

**Authors:** Elizabeth Parra Gatica, Gerardo Duran Ojeda, Michael Wendler

**Affiliations:** a Master Program in Dental Sciences, Faculty of Dentistry, Universidad de Concepción, Concepción, Chile; b Facultad de Ciencias de la Salud, Universidad Arturo Prat, Iquique, Chile; c Department of Restorative Dentistry, Faculty of Dentistry, Universidad de Concepción, Concepción, Chile

**Keywords:** Bulk fill, resin-based composite, flowable, degree of conversion, polymerization shrinkage

## Abstract

Flowable bulk-fill resin-based composites (BF-RBCs) represent a new and interesting alternative for the bulk-fill restorative techniques in the posterior region. However, they comprise a heterogeneous group of materials, with important differences in composition and design. Therefore, the aim of the present systematic review was to compare the main properties of flowable BF-RBCs, including their composition, degree of monomer conversion (DC), polymerization shrinkage and shrinkage stress, as well as flexural strength. The search was conducted following PRISMA guidelines in the Medline (PubMed), Scopus and Web of Science databases. *In vitro* articles reporting on the DC, polymerization shrinkage/shrinkage stress, and flexural strength of flowable BF-RBCs strength were included. The QUIN risk-of-bias (RoB) tool was used for assessing the study quality. From initially 684 found articles, 53 were included. Values for DC ranged between 19.41 and 93.71%, whereas polymerization shrinkage varied between 1.26 and 10.45%. Polymerization shrinkage stresses reported by most studies ranged between 2 and 3 MPa. Flexural strength was above 80 MPa for most materials. A moderate RoB was observed in most studies. Flowable BF-RBCs meet the requirements to be indicated for bulk fill restoration technique in the posterior region. However, important variations among composition and properties hinder extrapolation of the results to materials different from those reported here. Clinical studies are urgently required to assess their performance under a real working scenario.

## Introduction

Bulk-fill resin-based composites (BF-RBCs) were introduced to the dental market during the last decade, aiming to solve issues associated with the incremental technique in posterior teeth. Initially, the term bulk-fill was used to designate resin composites that allowed 4–5 mm thick increments, being adequate for the full-body and base bulk-fill techniques [1]. However, product marketing by dental manufacturers has slowly installed the bulk-fill concept as a new class of materials, rather than the technique to which it originally referred. This has become a non-negligible source of confusion among clinicians, as so-called BF-RBCs comprise an heterogenous group of materials, with important differences in composition and properties [2].

Resin composites aiming to be included into the BF-RBC category need to comply with two fundamental requirements: (1) an adequate degree of monomer conversion (DC) throughout the material; and (2) the ability to compensate or dissipate polymerization shrinkage stress at the cavity margins [2]. Whereas the former has been achieved to a large extent by increasing the translucency of the material and by using alternative and more efficient initiation systems than camphorquinone, [3] the latter has been optimized by modifying the chemistry of the monomers, as well as improving their interaction with the filler particles [4].

Current commercial BF-RBCs can be classified according to their viscosity in flowable (low viscosity) and sculptable (also high viscosity). Flowable RBCs have been known in the past to display inferior mechanical properties than high-viscosity RBCs, mainly due to the higher amount of filler particles in the latter [5]. Accordingly, their use was limited to small or minimally invasive cavity preparations, for the repair and sealing of defective restorations, and as cavity base or liner in larger restorations. However, incorporation of nanofillers to this new generation of flowable BF-RBCs has enhanced their mechanical properties while maintaining their low viscosity [6]. In addition, their self-leveling capacity guarantees an excellent adaptation to the cavity margins, [7] while displaying a high DC [8] and an improved stress relieving capacity [6,9]. Thus, they represent an interesting alternative for bulk-fill restorations in the posterior region, when combined with a final layer or cap of sculptable RBC [10].

To date, a large number of flowable BF-RBCs are available in the market. Despite their common seek for higher DC and reduced polymerization shrinkage stresses, manufacturers have gone different ways to achieve these goals, which in turn has led to different degrees of success. Accordingly, material selection and indication has become a difficult task for clinicians around the world. In this vein, the aim of the present systematic review was to compare, on the base of available scientific evidence, the main properties of flowable BF-RBCs, including their composition, DC, polymerization shrinkage and shrinkage stress, as well as flexural strength.

## Methods

This systematic review followed the recommendations of the Preferred Reporting Items for Systematic Reviews and Meta-Analyses (PRISMA) [11]. The systematic electronic search was conducted in the Medline (PubMed), Scopus and Web of Science (WOS) databases. The search strategy is presented in Table 1. Only articles written in English, with no more than 10 years of being published, reporting *in vitro* studies on the DC, polymerization shrinkage/shrinkage stress, and flexural strength of flowable BF-RBCs, were included. Systematic reviews and meta-analysis, articles written in any other language than English, as well as nonavailable full-text references were excluded. Studies that did not describe the exposure time to the light-curing unit or used any type of dual-curing BF-RBCs were also excluded. The inclusion criteria for each variable are summarized in Table 2. Regarding DC, only studies that evaluated polymerization using spectroscopic techniques and at 4 mm deep increments were included. For flexural strength, only studies using the 3-point-bending set-up, according to the ISO 4049, [12] were included. In the case of polymerization shrinkage, only studies determining volumetric contraction or its determination through microtomography, were included. Last search was conducted on 30^th^ of August 2022. Data were independently extracted by one reviewer (E.P.), and systematically classified in excel tables.

**Table 1.** Search strategy.

Pubmed	(((bulk-fill) OR (bulkfill)) AND ((flowable) OR (flow))) AND (“flexural strength”) (((bulk-fill) OR (bulkfill)) AND ((flowable) OR (flow))) AND (“polymerization shrinkage”) (((bulk-fill) OR (bulkfill)) AND ((flowable) OR (flow))) AND (((“polymerization stress”) OR (“polymerization shrinkage stress”)) OR (“shrinkage stress”)) (((bulk-fill) OR (bulkfill)) AND ((flowable) OR (flow))) AND (“degree of conversion”)
Scopus	TITLE-ABS-KEY ((((bulk-fill) OR (bulkfill)) AND ((flowable) OR (flow))) AND (“flexural strength”)) TITLE-ABS-KEY ((((bulk-fill) OR (bulkfill)) AND ((flowable) OR (flow))) AND (“polymerization shrinkage”)) TITLE-ABS-KEY ((((bulk-fill) OR (bulkfill)) AND ((flowable) OR (flow))) AND (((“polymerization stress”) OR (“polymerization shrinkage stress”)) OR (“shrinkage stress”))) TITLE-ABS-KEY ((((bulk-fill) OR (bulkfill)) AND ((flowable) OR (flow))) AND (“degree of conversion”))
Web of Science	TS=((((bulk-fill) OR (bulkfill)) AND ((flowable) OR (flow))) AND (“flexural strength”)) TS=((((bulk-fill) OR (bulkfill)) AND ((flowable) OR (flow))) AND (“polymerization shrinkage”)) TS=((((bulk-fill) OR (bulkfill)) AND ((flowable) OR (flow))) AND (((“polymerization stress”) OR (“polymerization shrinkage stress”)) OR (“shrinkage stress”))) TS=((((bulk-fill) OR (bulkfill)) AND ((flowable) OR (flow))) AND (((“polymerization stress”) OR (“polymerization shrinkage stress”)) OR (“shrinkage stress”)))

**Table 2.** Inclusion criteria.

**Degree of conversion **	Studies with spectroscopy-based evaluation methods and 4 mm depth samples.
**Polymerization shrinkage stress**	Studies that measure stress (MPa) and not force, through a universal testing machine or other device designed for this purpose.
**Polymerization shrinkage **	Studies measuring volumetric shrinkage (%), using microtomography as a method of evaluation.
**Flexural strength **	Studies measuring flexural strength (MPa) using the 3-point bending test.

The risk-of-bias (RoB) was assessed using the recently developed QUIN tool [13]. Briefly, 12 criteria, including aim/objective statement, sample size calculation, sampling technique, comparison group details, methodology explanation, operator details, randomization, outcome assessment and analysis, blinding, statistical analysis, and result presentation, were evaluated and rated according to ‘adequately specified’ (score = 2), ‘inadequately specified’ (score = 1), or ‘not specified’ (score = 0). Scores were subsequently added and the RoB of the study was estimated using: [13]

$$\mathrm{RoB}=\frac{total\;\mathrm{score}\;\times 100}{2\;\times \mathrm{applicable}\;\mathrm{criteria}}$$

Studies were then graded according to their RoB as high (<50%), medium (between 50 and 70%) or low risk (>70%).

## Results

**Figure 1.**
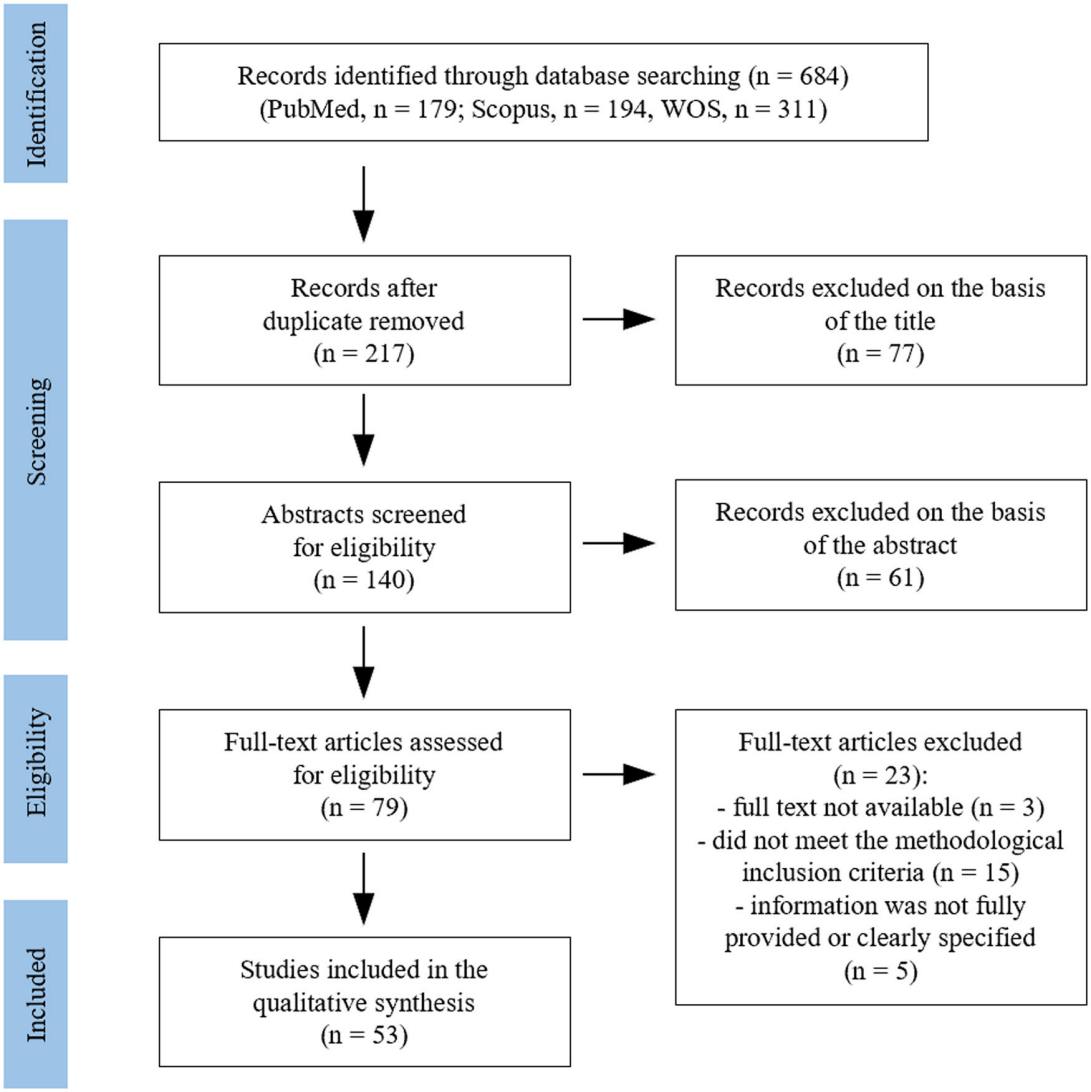
Flow diagram of the article identification and inclusion process.

Following duplicate removal (467), 217 eligible papers were identified (title selection). After abstract reading, 79 articles met the eligibility criteria, and their full texts were obtained and read. Subsequently, another 20 articles were excluded due to discrepancies in their methods and the inclusion criteria. Further three were excluded as no full text was available. The qualitative analysis was conducted on a total of 53 articles (Figure 1).

In total, 12 materials were studied in the included literature. The manufacturer’s information on their composition is listed in Table 3. Among selected articles, 22 evaluated the DC (Table 4), 9 the polymerization shrinkage (Table 5), 13 the polymerization shrinkage stress (Table 6) and 16 measured the flexural strength (Table 7) of one or more flowable BF-RBCs. In 6 of the included studies more than one property was simultaneously evaluated.

**Table 3.** Overview of resin composites and their composition according to manufacturers.

Material	Code	Resin Matrix	Filler	Load (% weight/% volume)
Filtek Bulk Fill Flow (3 M)	FBF	Bis-GMA, Bis-EMA, UDMA, procrylat resins	Combination of zirconia/silica and ytterbium trifluoride filler	64.5%/42.5%
SureFil SDR Flow (Dentsply)	SDR	Modified UDMA, EBPADMA, TEGDMA	Barium and Strontium alumino-fluoro-silicate glasses	68%/45%
Tetric EvoFlow Bulk Fill (Ivoclar-Vivadent)	TEBF	Bis-GMA, Bis-EMA,TCDDA	Ytterbium, Trifluoride, Barium aluminium silicate glass and copolymer	68.2%/46.4%
Beautifil Bulk Flowable (Shofu)	BBF	BisGMA, UDMA, Bis-MPEPP, TEGDMA	S-PRG filler based on fluoroboro aluminosilicate glass	72.5%/N.A.
Venus Bulk Fill (Kulzer)	VBF	UDMA,EBPDMA	Ba-Al-F-Si glass, SiO2, ytterbium trifluoride	65%/38%
X-tra base (Voco)	XTB	Aliphatic dimethacrylate, Bis-EMA	N.A.	75%/ N.A.
SDR Plus (Dentsply)	SDR+	Modified UDMA, TEGDMA	Barium and Strontium alumino-fluoro-silicate glasses, ytterbium fluoride;	70.5%/47.4%
Tetric Power Flow (Ivoclar-Vivadent)	TPF	Bis-GMA, TCDDA	barium glass, ytterbium trifluoride and copolymers	68.2%/46.4%
Bulk base /Bulk base Medium Flow/ Bulk base High Flow (Sun Medical)	BB/BBM/ BBH	N.A.	N.A.	N.A.
Filtek Fill and Core (3M)	FCF	N.A.	N.A.	N.A.
Estelite bulk fill flow (Tokuyama)	EBF	Bis-GMA, Bis-MPEPP, TEGMA	Supra Nano spherical SiO2-ZrO2	70%/56%
G-aenial Bulk Injectable (GC)	GBF	Bis EMA, UDMA	barium glass, silica	N.A.

Bis-GMA: bisphenol A diglycidyl ether dimethacrylate; BisEMA: ethoxylated bisphenol A glycol dimethacrylate; UDMA: urethane dimethacrylate; EBPADMA: Ethoxylated bisphenol A dimethacrylate; TEGDMA: triethyleneglycol dimethacrylate; TCDDA: tricyclodecane dimethanol diacrylate; Bis-MPEPP: Bisphenol A polyethoxy methacrylate; N.A.: not available.

**Table 4.** Degree of conversion main informed results.

Study	Materials	Degree of Conversion (%)
Al-Ahdal et al. *Dent Mater* 2015 https://doi.org/10.1016/j.dental.2015.07.004	FBF BBF VBF XTB	55.8–62.3^a^ 56.3–65.7^a^ 66.6–74.8^a^ 49.4–57.7^a^
Albuquerque et al. Eur J Gen Dent 2021 https://doi.org/10.4103/ejgd.ejgd_14_19	SDR	73.20/74.64^c^
Braga et al. *Oper Dent* 2019 https://doi.org/10.2341/17-351-L	SDR	64.6/75.6^b^
Czasch et al. *Clin Oral Investig* 2013 https://doi.org/10.1007/s00784-012-0702-8	SDR VBF	58.3–61.2^c^ 62.9–67.92^c^
Fronza et al. *Dent Mater* 2015 https://doi.org/10.1016/j.dental.2015.10.001	FBF SDR	75.3 81.2
Gonçalves et al. *Braz. Oral Res* 2018 https://doi.org/10.1590/1807-3107bor-2018.vol32.0017	FBF VBF	41.0 86.0
Harp et al. *J Esthet Restor Dent.* 2022 https://doi.org/10.1111/jerd.12901	SDR+ TEBF	60.2 47.5
Hayashi et al. *Dent Mater* 2020 https://doi.org/10.1016/j.dental.2020.10.012	TEBF TPF	58.7 60.1
Lempel et al. *Int J Mol Sci* 2016 https://doi.org/10.3390/ijms17050732	FBF SDR XTB	19.41/32.71^c^ 50.05^c^ 28.77/34.01^c^
Lempel et al. *Dent Mater* 2019 https://doi.org/10.1016/j.dental.2018.11.017	FBF SDR	36.7–49.2^c^ 42.8–63.0^c^
Lempel et al. *Dent Mater* 2021 https://doi.org/10.1016/j.dental.2021.02.013	SDR+	65
Majidinia et al. *Dental Hypotheses* 2020 https://doi.org/10.4103/denthyp.denthyp_41_19	FBF	63.06/66.28^c^
Marigo et al. *Eur Rev Med Pharmacol Sci* 2015	SDR	64.67
Marovic et al. *Acta Odontol Scand* 2015 https://doi.org/10.3109/00016357.2014.992810	SDR VBF XTB	70.0 78.8 67.5
Monterubbianesi et al. *Front Physiol* 2016 https://doi.org/10.3389/fphys.2016.00652	SDR	75.67–93.17^a,b^
Papadogiannis et al. *Dent Mater* 2015 http://doi.org/10.1016/j.dental.2015.09.022	SDR VBF XTB	63.20 58.49 45.83
Par et al. *Oper Dent* 2015 https://doi.org/10.2341/14-091-L	FBF SDR VBF XTB	63.4 74.7 83.3 66.3
Siagian et al. *J Contemp Dent Pract* 2020 https://doi.org/10.5005/jp-journals-10024-2848	SDR	41.76
Sousa-Lima et al. *Oper Dent* 2017 https://doi.org/10.2341/16-299-L	TEBF	85.7
Theobaldo et al. *Clin Cosmet Investig Dent* 2017 https://doi.org/10.2147/CCIDE.S130803	SDR	79.97–84.16^b,d^
Yu et al. *Oper Dent* 2017 https://doi.org/10.2341/16-027-L	SDR BBF	69.38 53.17
Zorzin et al. *Dent Mater* 2015 http://doi.org/10.1016/j.dental.2014.12.010	FBF SDR VBF XTB	66.4/69.26 52.04/72.18 80.07/73.93 62.53/61.53

^a^Different times post-cure. ^b^Different light curing units. ^c^Different curing times or modes ^d^Different preheating temperatures.

FBF: Filtek Bulk Fill Flow; SDR: SureFil SDR Flow; TEBF: Tetric EvoFlow Bulk Fill; BBF: Beautifil Bulk Flowable; VBF: Venus Bulk Fill; XTB: X-tra Base; SDR+: SDR Plus; TPF: Tetric Power Flow; EXF: EverX Flow; BB: Bulk Base; BBM: Bulk Base Medium Flow; BBH: Bulk Base High Flow; FCF: Filtek Fill and Core.

**Table 5.** Polymerization shrinkage main informed results.

Study	Materials	Polymerization shrinkage (%)
Algamaiah et al. *J Esthet Restor Dent* 2017 http://doi.org/10.1111/jerd.12275	FBF SDR	3.47/4.07^a^ 3.65/3.78^a^
Haugen et al. *Int J Mol Sci* 2020 http://doi.org/10.3390/ijms21145136	SDR FBF	3.36 3.39
Hirata et al. *Biomed Mater Res B Appl Biomater* 2015 http://doi.org/10.1002/jbm.b.33258	SDR	1.5/2.0^a^
Kamalak & Kamalak. *Biomed Res* 2018 https://doi.org/10.4066/biomedicalresearch.29-18-314	SDR FBF XTB	1.54 2.04 1.26
Oglakci et al. *J Adhes Sci Technol* 2020 https://doi.org/10.1080/01694243.2020.1782038	SDR+ EBF	4.4/10.41^b^ 4.07/10.45^b^
Prager et al. *Dent Mater J* 2018 https://doi.org/10.4012/dmj.2017-136	SDR VBF	6.55 4.07
Rizzante, Duque et al. *Dent Mater J* 2019 https://doi.org/10.4012/dmj.2018-063	FBF SDR XTB	3.34 3.36 3.11
Sampaio et al. *Oper Dent* 2017 https://doi.org/10.2341/15-296-L	FBF SDR	5.49 3.31
Sampaio et al. *Dent Mater* 2019 https://doi.org/10.1016/j.dental.2019.07.025	FBF TEBF	3.50 2.75

^a^Samples with/ without adhesive. ^b^Samples with/without thermo-mechanical ageing.

FBF: Filtek Bulk Fill Flow; SDR: SureFil SDR Flow; TEBF: Tetric EvoFlow Bulk Fill; BBF: Beautifil Bulk Flowable; VBF: Venus Bulk Fill; XTB: X-tra Base; SDR+: SDR Plus; TPF: Tetric Power Flow; EXF: EverX Flow; BB: Bulk Base; BBM: Bulk Base Medium Flow; BBH: Bulk Base High Flow; FCF: Filtek Fill and Core.

**Table 6.** Polymerization shrinkage stress informed results.

Study	Materials	Shrinkage stress (MPa)
Attik et al. *Dent Mater* 2022 https://doi.org/10.1016/j.dental.2021.12.029	SDR+	3.44
De Freitas Chaves et al. *J Conserv Dent* 2020 http://doi.org/10.4103/jcd.Jcd_58_19	FBF SDR XTB	19.45 24.24 18.68
Fronza et al. *J Adhes Dent* 2018 http://doi.org/10.2341/16-024-l	FBF	3.87
Fronza et al. *Dent Mater* 2015 http://doi.org/10.1016/j.dental.2015.10.001	FBF SDR	3.5 3.3
Han et al. *Oper Dent* 2017 http://doi.org/10.2341/16-023-L	SDR VBF	3.02 3.46
Han et al. *J Dent* 2019 https://doi.org/10.1016/j.jdent.2018.10.013	SDR	2.76
Kim et al. *J Dent* 2015 http://doi.org/10.1016/j.jdent.2015.02.002	FBF SDR	2.24 1.68
Kim et al. *Oper Dent* 2016 http://doi.org/10.2341/15-260-l	FBF SDR	2.28 1.70
Nakano et al. *Oper Dent* 2020 http://doi.org/10.2341/19-166-l	FBF TEBF	2.3 2.8
Rizzante et al. *J Appl Oral Sci* 2019 http://doi.org/10.1590/1678-7757-2018-0132	FBF SDR XTB	0.28/0.43^a^ 0.19/0.24^a^ 0.31/0.51^a^
Sousa-Lima et al. *Oper Dent* 2017 https://doi.org/10.2341/16-299-L	TEBF	0.77
Velo et al. *Braz Dent J* 2019 http://doi.org/10.1590/0103-6440201902571	FBF SDR	0.13 0.23
Zorzin et al. *Dent Mater* 2015 http://doi.org/10.1016/j.dental.2014.12.010	FBF SDR VBF XTB	1.55/1.47^b^ 1.33/ 1.29^b^ 1.65/ 1.74^b^ 1.45/ 1.68^b^

^a^Different sample volumes (12mm^3^/24mm^3^), ^b^Different curing times (manufacturer’s instructions/30s).

FBF: Filtek Bulk Fill Flow; SDR: SureFil SDR Flow; TEBF: Tetric EvoFlow Bulk Fill; BBF: Beautifil Bulk Flowable; VBF: Venus Bulk Fill; XTB: X-tra Base; SDR+: SDR Plus; TPF: Tetric Power Flow; EXF: EverX Flow; BB: Bulk Base; BBM: Bulk Base Medium Flow; BBH: Bulk Base High Flow; FCF: Filtek Fill and Core.

**Table 7.** Flexural strength main informed results.

Study	Materials	Flexural strength (MPa)
Alrahlah*. J Contemp Dent Pract* 2018 http://doi.org/10.1016/j.dental.2015.07.004	SDR	138.5/130.02^a^
Attik et al. *Dent Mater 2022* http://doi.org/10.1016/j.dental.2021.12.029	SDR+	115.7
Czasch et al. *Clin Oral Investig* 2013 http://doi.org/10.1007/s00784-012-0702-8	SDR VBF	131.8 122.7
de Freitas Chaves et al. *J Conserv Dent* 2020 http://doi.org/10.4103/jcd.Jcd_58_19	FBF SDR XTB	135.98 115.75 89.82
Gilli et al. *Oper Dent* 2022 http://doi.org/10.2341/21-084-L	FBF SDR VBF XTB	121.7 147.8 115.114 132.2
Hirokane et al. *Oper Dent* 2021 https://doi.org/10.2341/20-253-L	FBF BBF BBM SDR+ GBF	117.4/103.0^a^ 122.5/105.0^a^ 102.0/93.1^a^ 122.0/111.0^a^ 143.9/129.4^a^
Ilie et al. *Oper Dent* 2013 https://doi.org/10.2341/12-395-L	FBF SDR VBF XTB	122.4 131.8 122.7 139.4
Jung & Park. *Oper Dent* 2017 https://doi.org/10.2341/16-254-L	SDR VBF	101.26 97.36
Lassila et al. *Odontology* 2019 https://doi.org/10.1007/s10266-018-0405-y	FBF SDR TEBF	122 120 97
Leprince et al. *J Dent* 2014 http://doi.org/10.1016/j.jdent.2014.05.009	FBF SDR VBF XTB	88.4 100.2 76.0 110.5
Nitta et al. *Dent Mater J* 2017 http://doi.org/10.4012/dmj.2016-394	FBF ^a^BBM ^a^BBH	132.8 96.2 89
Öznurhan et al. *J Clin Pediatr Dent* 2015 http://doi.org/10.17796/1053-4628-39.3.241	SDR XTB	45.0 40.96
Shimatani et al. *Oper Dent* 2020 https://doi.org/10.2341/18-160-L	SDR XTB BBF BB FCF	105.7 110.4 102.1 68.9 116.1
Sousa-Lima et al. *Oper Dent* 2017 https://doi.org/10.2341/16-299-L	TEBF	76.6
Tsujimoto et al. *Polymers* 2021 https://doi.org/10.3390/polym13162613	FBF	50.3/144.9^b^
Oh et al. *Biomater Res 2022* https://doi.org/10.1186/s40824-022-00267-5	SDR	127.69 − 135.83^c^

^a^Samples with/without thermo-mechanical ageing. ^b^Different times post-cure. ^c^Different irradiation distance.

FBF: Filtek Bulk Fill Flow; SDR: SureFil SDR Flow; TEBF: Tetric EvoFlow Bulk Fill; BBF: Beautifil Bulk Flowable; VBF: Venus Bulk Fill; XTB: X-tra Base; SDR+: SDR Plus; TPF: Tetric Power Flow; EXF: EverX Flow; BB: Bulk Base; BBM: Bulk Base Medium Flow; BBH: Bulk Base High Flow; FCF: Filtek Fill and Core; GBF: G-aenial Bulk Injectable.

**Table 8.** Quality assessment of risk of bias of *in vitro* studies (QUIN tool).

Study	Risk of Bias	Study grading
Al-Ahdal K et al. *Dent Mater* 2015 https://doi.org/10.1016/j.dental.2015.07.004	58.33%	Medium risk
Albuquerque et al. *Eur J Gen Dent* 2021 https://doi.org/10.4103/ejgd.ejgd_14_19	58.33%	Medium risk
Alrahlah et al. *J Contemp Dent Pract* 2018 https://doi.org/10.5005/jp-journals-10024-2205	58.33%	Medium risk
Algamaiah et al. *J Esthet Restor Dent* 2016 https://doi.org/10.1111/jerd.12275	66.67%	Medium risk
Attik et al. *Dent Mater* 2022 https://doi.org/10.1016/j.dental.2021.12.029	58.33%	Medium risk
Braga et al. *Oper Dent* 2019 https://doi.org/10.2341/17-351-L	62.5%	Medium risk
Czasch et al. *Clin Oral Investig* 2013 https://doi.org/10.1007/s00784-012-0702-8	54.17%	Medium risk
De Freitas et al. *J Conserv Dent* 2020 https://doi.org/10.4103/JCD.JCD_58_19	62.5%	Medium risk
Fronza et al. *Dent Mater* 2015 https://doi.org/10.1016/j.dental.2015.10.001	66.67%	Medium risk
Fronza et al. *J Adhes Dent* 2018 https://doi.org/10.3290/j.jad.a40987	54.17%	Medium risk
Gilli et al. *Oper Dent* 2022 http://doi.org/10.2341/21-084-L	58.33%	Medium risk
Gonçalvez et al. *Braz Oral Res* 2018 https://doi.org/10.1590/1807-3107bor-2018.vol32.0017	58.33%	Medium risk
Han et al. *Oper Dent* 2017 https://doi.org/10.2341/16-023-L	70.83%	Low risk
Han et al. *J Dent* 2019 https://doi.org/10.1016/j.jdent.2018.10.013	70.83%	Low risk
Harp et al. *J Esthet Restor Dent* 2022 https://doi.org/10.1111/jerd.12901	62.50%	Medium risk
Haugen et al. *Int J Mol Sci* 2020 https://doi.org/10.3390/ijms21145136	62.5%	Medium risk
Hayashi et al. *Dent Mater* 2020 https://doi.org/10.1016/j.dental.2020.10.012	58.33%	Medium risk
Hirata et al. *Biomed Mater Res B Appl Biomater* 2014 https://doi.org/10.1002/jbm.b.33258	54.17%	Medium risk
Hirokane et al. *Oper Dent* 2021 https://doi.org/10.2341/20-253-L	66.67%	Medium risk
Ilie et al. *Oper Dent* 2013 https://doi.org/10.2341/12-395-L	58.33%	Medium risk
Jung & Park *Oper Dent* 2017 https://doi.org/10.2341/16-254-L	58.33%	Medium risk
Kamalak & Kamalak *Biomed Res* 2018 10.4066/biomedicalresearch.29-18-314	50%	High risk
Kim et al. *J Dent* 2015 https://doi.org/10.1016/j.jdent.2015.02.002	58.33%	Medium risk
Kim et al. *Oper Dent* 2016 https://doi.org/10.2341/15-260-L	58.33%	Medium risk
Lassila et al. *Odontology* 2019 https://doi.org/10.1007/s10266-018-0405-y	58.33%	Medium risk
Lampel et al. *Dent Mater* 2021 https://doi.org/10.1016/j.dental.2021.02.013	58.33%	Medium risk
Lempel et al. *Int J Mol Sci* 2016 https://doi.org/10.3390/ijms17050732	62.5%	Medium risk
Lempel et al. *Dent Mater* 2019 https://doi.org/10.1016/j.dental.2018.11.017	62.5%	Medium risk
Leprince et al. *J Dent* 2014 https://doi.org/10.1016/j.jdent.2014.05.009	54.17%	Medium risk
Majidinia et al. *Dental Hypotheses* 2020 https://doi.org/10.4103/denthyp.denthyp_41_19	58.33%	Medium risk
Marigo et al. *Eur Rev Med Pharmacol Sci* 2015	58.33%	Medium risk
Marovic et al. *Acta Odontol Scand* 2014 https://doi.org/10.3109/00016357.2014.992810	62.5%	Medium risk
Monterubbianesi et al. *Front Physiol* 2016 https://doi.org/10.3389/fphys.2016.00652	58.33%	Medium risk
Nakano et al. *Oper Dent* 2020 https://doi.org/10.2341/19-166-L	54.17%	Medium risk
Nitta et al. *Dent Mater J* 2017 https://doi.org/10.4012/dmj.2016-394	58.33%	Medium risk
Oglakci et al. *J Adhes Sci Technol* 2020 https://doi.org/10.1080/01694243.2020.1782038	62.50%	Medium risk
Oh et al. *Biomater Res* 2022 https://doi.org/10.1186/s40824-022-00267-5	58.33%	Medium risk
Öznurhan et al. *J Clin Pediatr Dent* 2015 https://doi.org/10.17796/1053-4628-39.3.241	54.17%	Medium risk
Papadogiannis et al. *Dent Mater* 2015 https://doi.org/10.1016/j.dental.2015.09.022	54.17%	Medium risk
Par et al. *Oper Dent* 2015 https://doi.org/10.2341/14-091-L	58.33%	Medium risk
Prager et al. *Dent Mater J* 2018 https://doi.org/10.4012/dmj.2017-136	58.33%	Medium risk
Rizzante et al. *J Appl Oral Sci* 2019 https://doi.org/10.1590/1678-7757-2018-0132	54.17%	Medium risk
Rizzante et al. *Dent Mater J* 2019 https://doi.org/10.4012/dmj.2018-063	54.17%	Medium risk
Sampaio et al. *Oper Dent* 2016 https://doi.org/10.2341/15-296-L	70.83%	Low risk
Sampaio et al. *Dent Mater* 2019 https://doi.org/10.1016/j.dental.2019.07.025	70.83%	Low risk
Shimatani et al. *Oper Dent* 2020 https://doi.org/10.2341/18-160-L	62.5%	Medium risk
Siagian et al. *J Contemp Dent Pract* 2020 https://doi.org/10.5005/jp-journals-10024-2848	54.17%	Medium risk
Sousa-Lima et al. *Oper Dent* 2017 https://doi.org/10.2341/16-299-L	58.33%	Medium risk
Theobaldo et al. *Clin Cosmet Investig Dent* 2017 https://doi.org/10.2147/CCIDE.S130803	62.5%	Medium risk
Tsujimoto et al. *Polymers* 2021 https://doi.org/10.3390/polym13162613	54.17%	Medium risk
Velo et al. *Braz Dent J* 2019 https://doi.org/10.1590/0103-6440201902571	62.5%	Medium risk
Yu et al. *Oper Dent* 2017 https://doi.org/10.2341/16-027-L	54.17%	Medium risk
Zorzin et al. *Dent Mater* 2015 https://doi.org/10.1016/j.dental.2014.12.010	58.33%	Medium risk

RoB <50% high risk; 50%<RoB < 70% medium risk; RoB > 70% low risk.

A high variability was found for the DC of flowable BF-RBCs in the current systematic review (Table 4). Whereas the highest DC values were reported for Venus Bulk Fill and Surefil SDR Flow, which displayed a 93.71%, [14] the lowest DC was measured for Filtek Bulk Fill Flowable, which reached only a 32.71% in the study by Lempel et al. [8]. An inverse relation was observed between the DC and the polymerization depth, with most studies reporting DC values above 55% at distances below 4 mm from the light source (Table 4).

Polymerization shrinkage reported by the studies included in this systematic review ranged between 1.26% and 10.45%, although most of them were around 3.5% (Table 5). A similar volume shrinkage value was reported by two of the studies for the materials SureFil SDR Flow and Filtek Bulk Fill Flow, [15,16] despite the slightly lower filler fraction of the latter (Table 5). However, a significantly higher value was measured by Sampaio et al. for Filtek Bulk Fill Flow when applied in class I cavities [9]. A similar trend was observed by the same authors for Tetric EvoFlow Bulk Fill when compared to Filtek Bulk Fill Flow, [17] pointing to an inverse relationship between the filler content and the polymerization shrinkage of these materials.

A high variation was also observed for the polymerization shrinkage stress (Table 6), with the lowest informed value as low as 0.13 MPa [18] and the highest reaching up 24.24 MPa [19]. Still, most studies reported values at the lower end of this span, between 2 and 3 MPa (Table 6). Statistically significant differences between materials were observed in most studies, with SureFil SDR Flow outperforming Filtek Bulk Fill Flow in four out of seven studies, [7,20–22] and Venus Bulk Fill in other two studies [22,23].

Most studies included in this systematic review reported mean flexural strength values above 80 MPa (Table 7), fulfilling the requirements of the ISO 4049 standard [12] for type-1 resin composite materials. Among the reported flowable BF-RBC, the highest values were obtained by X-tra base (139.4 MPa) [24], G-aenial Bulk Injectable (143.9 MPa) [25] and Filtek Bulk Fill Flow (144.9 MPa) [26]. On the other hand, only four studies reported flexural strength values below the 80 MPa threshold [6,26–28].

Regarding the RoB analysis, only four studies were graded as ‘low risk’, whereas one study was categorized as ‘high risk’ and the remaining 48 had a medium bias risk (Table 8). Most studies displayed an inadequate description of the sample size calculation, control group/s, as well as operator details, calibration processes and blinding (for more details, please refer to Table S1 in the Supplemental Material).

## Discussion

The overall quality of the studies was rated with a moderate risk of bias. This implied in most studies that part of the information regarding sampling, test conduction and blinding was missing or incorrectly reported (see Table S1 in the Supplemental Material). Guidelines for reporting *in vitro* studies are still a pending matter in the dental field, an issue that has been thoroughly worked out in clinical and epidemiological studies, as well as in case reports and systematic reviews. Development of instruments to assess RoB, like the QUIN tool [13] used here or the recently published RoBDEMAT, [29] represent an interesting start point to improve the design and communication quality of *in vitro* studies.

Although to date no consensus has been reached about a minimum value for DC, an increase in the monomer conversion of the material has been often associated with improved mechanical properties [14,30,31]. In addition, a low DC has been associated with increased microleakage, [32] marginal breakdown, [33] as well as lower hardness and wear resistance [33]. Critical values for these parameters seem to occur when the DC is below 55–65%, which has been proposed as a monomer conversion threshold in the past [34,35]. Regarding laboratory methods to determine the polymerization efficiency of RBCs, different alternatives have been proposed, including indirect techniques (such as hardness or nanoindentation), as well as direct methods (mainly vibrational spectroscopy). In the present review, only articles that conducted a direct evaluation of the material’s DC were included, as these techniques base on the quantification of the non-reactive C = C bonds, [36] displaying higher precision than their indirect counterparts. Among them, Fourier-transform infrared spectroscopy (FTIR), as well as Raman spectroscopy, are the most common and widely used methods.

Observed differences in the DC of the flowable BF-RBCs reported here (Table 4) have been related to their intrinsic characteristics (i.e. photoinitiator system, resin matrix chemistry, filler type, etc.), as well as with external factors, including the restorative technique (increment thickness, application temperature), light activation (curing mode, exposure time) and the light-curing unit itself (light intensity, wavelength, heat emission, diameter, etc.) [37]. Accordingly, different strategies have been followed by the manufacturers to achieve satisfactory conversion degrees in their materials. Materials that incorporate monomers with lower viscosity (Bis-EMA, UDMA), have shown to increase their DC when compared to RBCs that have only Bis-GMA and TEGDMA in their composition [38]. In the case of Venus Bulk Fill, its high translucency and low filler content increase light transmission through the material, favoring photoinitiation in deeper increments [39]. Similarly, SureFil SDR Flow incorporates a lesser amount of filler content, but with an increased particle size, which further decreases light scattering in the material [40]. In addition, a photo-active group is embedded in the UDMA monomers, aiming to optimize the polymerization process thorough interaction with camphorquinone [41]. The manufacturer of Tetric EvoFlow Bulk Fill, on the other hand, switched to a different photoinitiator system, based on benzoyl germanium derivates (under the commercial trade name Ivocerin), which has shown a higher photoinitiation activity and thus, a higher efficiency at lower light intensities than its camphorquinone counterpart [42].

Average values reported for the polymerization shrinkage of flowable BF-RBCs (Table 5) match previously reported data for conventional paste BF-RBCs (2.0 − 3.5%) [15,17,21,43] and are below conventional flowable RBCs (4.00 − 5.50%) [44]. Although it may be intuitive to expect lower polymerization shrinkage stresses in materials with a low-volume shrinkage, it has been shown that both properties are not always directly related [44]. The low polymerization shrinkage stresses reported by most studies included in this systematic review (Table 6) seem to confirm this trend, underlining the effectiveness of stress-control mechanisms introduced by the manufacturers. Furthermore, the few studies that looked at both properties found polymerization shrinkage stresses in the range of 1 to 2.5 MPa even in flowable BF-RBCs with polymerization shrinkage exceeding 4% [20,22,45].

It has been suggested that the replacement of higher molecular weight monomers (such as UDMA) by others with lower molecular weight (e.g. Bis-EMA), may contribute to limit volumetric reduction, and thus polymerization shrinkage stress [6]. Incorporation of stress releasing monomers, on the other hand, as well as the use of so-called ‘intelligent fillers’, can increase the flexibility of the chains during the pre-gelation phase, compensating stress development at the adhesive interfaces [20,45]. These effects seem to add to each other, rendering a general reduction of the polymerization shrinkage stress in the reported materials. This may imply that the use of these materials in large increments does not represent an important risk for the integrity of the adhesive interfaces of the restorations.

Manufacturer’s recommendations of most commercially available flowable BF-RBCs is to cover the restoration with a final layer of conventional resin composite [10]. This allows to compensate to some extent its lower flexural strength, [6] as well as to prevent accelerated wear of the restoration. This balance becomes critical in extensive tooth preparations that are exposed to high mechanical loads, since the mechanical performance of the restoration could be compromised due to an excessive volume of the base material. Nevertheless, results of the present systematic review revealed that the flexural strength of most flowable BF-RBCs were above the threshold defined by the ISO 4049 standard [12]. In addition, studies that also included in their experimental design high viscosity BF-RBCs found strength values in the same range of those displayed by the flowable BF-RBC [24,27,46,47]. For instance, no statistical differences in the flexural strength of X-tra Base (flowable) and X-tra fill (sculptable) were measured by Ilie et al. [24] as well as by Leprince et al. [27] despite their difference in filler content (75 vs. 86 wt.%). This highlights the reduction in the gap between the mechanical properties of the two material categories, encouraging the use of low viscosity RBCs for the fill-up of large and deep cavities.

## Conclusion

Results of this systematic review highlight the good standard achieved by contemporary flowable BF-RBCs. In terms of DC, flexural strength and polymerization shrinkage/shrinkage stress, they meet necessary requirements to be indicated for the bulk-fill restoration techniques in the posterior region. However, important variations among composition and design of this “new” class of RBCs hinder extrapolation of the results to materials different from those reported here. In addition, clinical studies are urgently required to assess their performance under a real working scenario.

## Supplementary Material

Supplemental Material

## References

[1] Corral-Núnez C, Vildósola-Grez P, Bersezio-Miranda C, et al. Sate of the art of bulk-fill resin-based composites: a review. Revista Facultad de Odontología Universidad de Antioquia. 2015;27:177–196.

[2] Van Ende A, De Munck J, Lise DP, et al. Bulk-Fill composites: a review of the current literature. J Adhes Dent. 2017;19(2):95–109. 28443833 10.3290/j.jad.a38141

[3] Ilie N, Schöner C, Bücher K, et al. An in-vitro assessment of the shear bond strength of bulk-fill resin composites to permanent and deciduous teeth. J Dent. 2014;42(7):850–855. 24704081 10.1016/j.jdent.2014.03.013

[4] Fronza BM, Rueggeberg FA, Braga RR, et al. Monomer conversion, microhardness, internal marginal adaptation, and shrinkage stress of bulk-fill resin composites. Dent Mater. 2015;31(12):1542–1551. 26608118 10.1016/j.dental.2015.10.001

[5] Ferracane JL. Resin composite–state of the art. Dent Mater. 2011;27(1):29–38. 21093034 10.1016/j.dental.2010.10.020

[6] Sousa-Lima RX, Silva L, Chaves L, et al. Extensive assessment of the physical, mechanical, and adhesion behavior of a low-viscosity bulk fill composite and a traditional resin composite in tooth cavities. Oper Dent. 2017;42(5):E159–e66. 28829935 10.2341/16-299-L

[7] Kim RJ, Kim YJ, Choi NS, et al. Polymerization shrinkage, modulus, and shrinkage stress related to tooth-restoration interfacial debonding in bulk-fill composites. J Dent. 2015;43(4):430–439. 25676178 10.1016/j.jdent.2015.02.002

[8] Lempel E, Czibulya Z, Kovács B, et al. Degree of conversion and BisGMA, TEGDMA, UDMA elution from flowable bulk fill composites. IJMS. 2016;17(5):732. 27213361 10.3390/ijms17050732 PMC4881554

[9] Sampaio CS, Chiu KJ, Farrokhmanesh E, et al. Microcomputed tomography evaluation of polymerization shrinkage of class I flowable resin composite restorations. Oper Dent. 2017;42(1):E16–e23. 27689769 10.2341/15-296-L

[10] Hirata R, Kabbach W, de Andrade OS, et al. Bulk fill composites: an anatomic sculpting technique. J Esthet Restor Dent. 2015;27(6):335–343. 26177219 10.1111/jerd.12159

[11] Moher D, Liberati A, Tetzlaff J, PRISMA Group, et al. Preferred reporting items for systematic reviews and meta-analyses: the PRISMA statement. Int J Surg. 2010;8(5):336–341. 20171303 10.1016/j.ijsu.2010.02.007

[12] ISO 4049:2019(E) Dentistry-polymer-based filling, restorative and luting materials. 2019.

[13] Sheth VH, Shah NP, Jain R, et al. Development and validation of a risk-of-bias tool for assessing in vitro studies conducted in dentistry: the QUIN. J Prosthetic Dent. 2022; 10.1016/j.prosdent.2022.05.019. 35752496

[14] Monterubbianesi R, Orsini G, Tosi G, et al. Spectroscopic and mechanical properties of a new generation of bulk fill composites. Front Physiol. 2016;7:652. 28082918 10.3389/fphys.2016.00652 PMC5186780

[15] Algamaiah H, Sampaio CS, Rigo LC, et al. Microcomputed tomography evaluation of volumetric shrinkage of Bulk-Fill composites in class II cavities. J Esthet Restor Dent. 2017;29(2):118–127. 27925387 10.1111/jerd.12275

[16] Rizzante FAP, Duque JA, Duarte MAH, et al. Polymerization shrinkage, microhardness and depth of cure of bulk fill resin composites. Dent Mater J. 2019;38(3):403–410. 30918231 10.4012/dmj.2018-063

[17] Sampaio CS, Fernández Arias J, Atria PJ, et al. Volumetric polymerization shrinkage and its comparison to internal adaptation in bulk fill and conventional composites: a μCT and OCT in vitro analysis. Dent Mater. 2019;35(11):1568–1575. 31500903 10.1016/j.dental.2019.07.025

[18] Velo M, Wang L, Furuse AY, et al. Influence of modulated photo-activation on shrinkage stress and degree of conversion of bulk-Fill composites. Braz Dent J. 2019;30(6):592–598. 31800754 10.1590/0103-6440201902571

[19] de Freitas Chaves LV, de Sousa Lima RX, de Azevedo Silva LJ, et al. Bonding performance and mechanical properties of flowable bulk-fill and traditional composites in high c-factor cavity models. J Conserv Dent. 2020;23(1):36–41. 33223639 10.4103/JCD.JCD_58_19 PMC7657427

[20] Kim YJ, Kim R, Ferracane JL, et al. Influence of the compliance and layering method on the wall deflection of simulated cavities in bulk-fill. Oper Dent. 2016;41(6):e183–e94. 27820696 10.2341/15-260-L

[21] Rizzante FAP, Mondelli RFL, Furuse AY, et al. Shrinkage stress and elastic modulus assessment of bulk-fill composites. J Appl Oral Sci. 2019;27:e20180132. 30624465 10.1590/1678-7757-2018-0132 PMC6322642

[22] Zorzin J, Maier E, Harre S, et al. Bulk-fill resin composites: polymerization properties and extended light curing. Dent Mater. 2015;31(3):293–301. 25582061 10.1016/j.dental.2014.12.010

[23] Han SH, Park SH. Comparison of internal adaptation in class II bulk-fill composite restorations using Micro-CT. Oper Dent. 2017;42(2):203–214. 27892836 10.2341/16-023-L

[24] Ilie N, Bucuta S, Draenert M. Bulk-fill resin-based composites: an in vitro assessment of their mechanical performance. Oper Dent. 2013;38(6):618–625. 23570302 10.2341/12-395-L

[25] Hirokane E, Takamizawa T, Tamura T, et al. Handling and mechanical properties of low-viscosity bulk-fill resin composites. Oper Dent. 2021;46(5):E185–E198. 35486512 10.2341/20-253-L

[26] Tsujimoto A, Irie M, Teixeira ECN, et al. Relationships between flexural and bonding properties, marginal adaptation, and polymerization shrinkage in flowable composite restorations for dental application. Polymers. 2021;13(16):2613. 34451153 10.3390/polym13162613 PMC8398176

[27] Leprince JG, Palin WM, Vanacker J, et al. Physico-mechanical characteristics of commercially available bulk-fill composites. J Dent. 2014;42(8):993–1000. 24874951 10.1016/j.jdent.2014.05.009

[28] Shimatani Y, Tsujimoto A, Barkmeier WW, et al. Simulated cuspal deflection and flexural properties of Bulk-Fill and conventional flowable resin composites. Oper Dent. 2020;45(5):537–546. 32216724 10.2341/18-160-L

[29] Delgado AHS, Sauro S, Lima AF, et al. RoBDEMAT: a risk of bias tool and guideline to support reporting of pre-clinical dental materials research and assessment of systematic reviews. J Dent. 2022;127:104350. 36341980 10.1016/j.jdent.2022.104350

[30] Lovell LG, Lu H, Elliott JE, et al. The effect of cure rate on the mechanical properties of dental resins. Dent Mater. 2001;17(6):504–511. 11567688 10.1016/s0109-5641(01)00010-0

[31] Sarma A, Nagar P. A comparative evaluation of time-dependent changes on the surface hardness of bulk cure composites: an in vitro study. Int J Clin Pediatr Dent. 2018;11(3):183–187. 30131638 10.5005/jp-journals-10005-1508 PMC6102429

[32] Bruschi Alonso RC, Carvalho de Souza-Júnior EJ, Dressano D, et al. Effect of photoinitiator concentration on marginal and internal adaptation of experimental composite blends photocured by modulated methods. Eur J Dent. 2013;7(Suppl 1):S001–S008. 24966715 10.4103/1305-7456.119056 PMC4054066

[33] Ferracane JL, Mitchem JC, Condon JR, et al. Wear and marginal breakdown of composites with various degrees of cure. J Dent Res. 1997;76(8):1508–1516. 9240388 10.1177/00220345970760081401

[34] Silikas N, Eliades G, Watts DC. Light intensity effects on resin-composite degree of conversion and shrinkage strain. Dent Mater. 2000;16(4):292–296. 10831785 10.1016/s0109-5641(00)00020-8

[35] Soares LE, Liporoni PC, Martin AA. The effect of soft-start polymerization by second generation LEDs on the degree of conversion of resin composite. Oper Dent. 2007;32(2):160–165. 17427825 10.2341/06-45

[36] Shin WS, Li XF, Schwartz B, et al. Determination of the degree of cure of dental resins using raman and FT-Raman spectroscopy. Dent Mater. 1993;9(5):317–324. 7995484 10.1016/0109-5641(93)90050-z

[37] Reis AF, Vestphal M, Amaral RCD, et al. Efficiency of polymerization of bulk-fill composite resins: a systematic review. Brazilean Oral Res. 2017;31(suppl 1):e59. 10.1590/1807-3107BOR-2017.vol31.0059 28902239

[38] Tarle Z, Attin T, Marovic D, et al. Influence of irradiation time on subsurface degree of conversion and microhardness of high-viscosity bulk-fill resin composites. Clin Oral Invest. 2015;19(4):831–840. 10.1007/s00784-014-1302-6 25138041

[39] Goncalves F, Campos LMP, Rodrigues-Junior EC, et al. A comparative study of bulk-fill composites: degree of conversion, post-gel shrinkage and cytotoxicity. Brazilean Oral Res. 2018;32:e17. 10.1590/1807-3107bor-2018.vol32.0017 29538479

[40] Fronza BM, Ayres A, Pacheco RR, et al. Characterization of inorganic filler content, mechanical properties, and light transmission of bulk-fill resin composites. Oper Dent. 2017;42(4):445–455. 28402731 10.2341/16-024-L

[41] Li X, Pongprueksa P, Van Meerbeek B, et al. Curing profile of bulk-fill resin-based composites. J Dent. 2015;43(6):664–672. 25597265 10.1016/j.jdent.2015.01.002

[42] Moszner N, Fischer UK, Ganster B, et al. Benzoyl germanium derivatives as novel visible light photoinitiators for dental materials. Dent Mater. 2008;24(7):901–907. 18155290 10.1016/j.dental.2007.11.004

[43] Hirata R, Clozza E, Giannini M, et al. Shrinkage assessment of low shrinkage composites using micro-computed tomography. J Biomed Mater Res B Appl Biomater. 2015;103(4):798–806. 25115608 10.1002/jbm.b.33258

[44] Kleverlaan CJ, Feilzer AJ. Polymerization shrinkage and contraction stress of dental resin composites. Dent Mater. 2005;21(12):1150–1157. 16040118 10.1016/j.dental.2005.02.004

[45] Nakano EL, de Souza A, Boaro L, et al. Polymerization stress and gap formation of self-adhesive, bulk-fill and flowable composite resins. Oper Dent. 2020;45(6):E308–e16. 32516396 10.2341/19-166-L

[46] Jung JH, Park SH. Comparison of polymerization shrinkage, physical properties, and marginal adaptation of flowable and restorative bulk fill Resin-Based. Oper Dent. 2017;42(4):375–386. 28402737 10.2341/16-254-L

[47] Öznurhan F, Ünal M, Kapdan A, et al. Flexural and microtensile bond strength of bulk fill materials. J Clin Pediatr Dent. 2015;39(3):241–246. 26208069 10.17796/1053-4628-39.3.241

