# Supplementary material for: Contemporary flowable bulk-fill resin-based composites: a systematic review
Source: Biomater Investig Dent. 2023 Feb 22;10(1):8–19. doi: 10.1080/26415275.2023.2175685 (PMC10150621; doi:10.1080/26415275.2023.2175685)
Supplement: Supplemental Material [file IABO_A_2175685_SM5737.docx]

**Quality assessment of risk of bias of in vitro studies (QUIN tool)**

|  | Article | C1 | C2 | C3 | C4 | C5 | C6 | C7 | C8 | C9 | C10 | C11 | C12 | TS | %RoB | Low risk | Medium risk | High risk |
| --- | --- | --- | --- | --- | --- | --- | --- | --- | --- | --- | --- | --- | --- | --- | --- | --- | --- | --- |
| 1 | Al-Ahdal K, 2015  <http://dx.doi.org/10.1016/j.dental.2015.07.004> | 2 | 1 | 2 | 1 | 2 | 0 | 0 | 2 | 0 | 0 | 2 | 2 | 14 | 58,33% |  | Medium risk |  |
| 2 | Albuquerque F, 2021  <https://doi.org/10.4103/ejgd.ejgd_14_19> | 2 | 1 | 2 | 1 | 2 | 0 | 0 | 2 | 0 | 0 | 2 | 2 | 14 | 58,33% |  | Medium risk |  |
| 3 | Ali Alrahlah, 2018  <https://doi.org/10.5005/jp-journals-10024-2205> | 2 | 1 | 2 | 1 | 2 | 0 | 0 | 2 | 0 | 0 | 2 | 2 | 14 | 58,33% |  | Medium risk |  |
| 4 | Algamaiah H, 2016  <https://doi.org/10.1111/jerd.12275> | 2 | 1 | 2 | 1 | 2 | 0 | 2 | 2 | 0 | 0 | 2 | 2 | 16 | 66,67% |  | Medium risk |  |
| 5 | Attik N, 2022  <https://doi.org/10.1016/j.dental.2021.12.029> | 2 | 1 | 2 | 1 | 2 | 0 | 0 | 2 | 0 | 0 | 2 | 2 | 14 | 58,33% |  | Medium risk |  |
| 6 | Braga S, 2019  <https://doi.org/10.2341/17-351-L> | 2 | 1 | 2 | 1 | 2 | 1 | 0 | 2 | 0 | 0 | 2 | 2 | 15 | 62,5% |  | Medium risk |  |
| 7 | Czasch P, 2013  <https://doi.org/10.1007/s00784-012-0702-8> | 1 | 1 | 2 | 1 | 2 | 0 | 0 | 2 | 0 | 0 | 2 | 2 | 13 | 54,17% |  | Medium risk |  |
| 8 | de Freitas Chaves LV, 2020  <https://doi.org/10.4103/JCD.JCD_58_19> | 2 | 1 | 2 | 1 | 2 | 0 | 2 | 2 | 0 | 0 | 1 | 2 | 15 | 62,5% |  | Medium risk |  |
| 9 | Fronza B, 2015  <http://dx.doi.org/10.1016/j.dental.2015.10.001> | 2 | 1 | 2 | 2 | 2 | 0 | 2 | 2 | 0 | 0 | 1 | 2 | 16 | 66,67% |  | Medium risk |  |
| 10 | Fronza, 2018  <https://doi.org/10.3290/j.jad.a40987> | 2 | 1 | 2 | 1 | 2 | 0 | 0 | 2 | 0 | 0 | 1 | 2 | 13 | 54,17% |  | Medium risk |  |
| 11 | Gilli M, 2022  <http://doi.org/10.2341/21-084-L> | 2 | 1 | 2 | 1 | 2 | 0 | 0 | 2 | 0 | 0 | 2 | 2 | 14 | 58,33% |  | Medium risk |  |
| 12 | Gonçalvez F, 2017  <https://doi.org/10.1590/1807-3107bor-2018.vol32.0017> | 2 | 1 | 2 | 1 | 2 | 0 | 0 | 2 | 0 | 0 | 2 | 2 | 14 | 58,33% |  | Medium risk |  |
| 13 | Han SH, 2017  <https://doi.org/10.2341/16-023-L> | 2 | 1 | 2 | 2 | 2 | 0 | 2 | 2 | 0 | 0 | 2 | 2 | 17 | 70,83% | Low risk |  |  |
| 14 | Han SH, 2019  <https://doi.org/10.1016/j.jdent.2018.10.013> | 2 | 1 | 2 | 2 | 2 | 0 | 2 | 2 | 0 | 0 | 2 | 2 | 17 | 70,83% | Low risk |  |  |
| 15 | Harp Y, 2022  <https://doi.org/10.1111/jerd.12901> | 1 | 1 | 2 | 1 | 2 | 0 | 2 | 2 | 0 | 0 | 2 | 2 | 15 | 62,50% |  | Medium risk |  |
| 16 | Haugen H, 2020  <https://doi.org/10.3390/ijms21145136> | 2 | 1 | 2 | 2 | 2 | 0 | 0 | 2 | 0 | 0 | 2 | 2 | 15 | 62,5% |  | Medium risk |  |
| 17 | Hayashi J, 2020  <https://doi.org/10.1016/j.dental.2020.10.012> | 2 | 1 | 2 | 1 | 2 | 0 | 0 | 2 | 0 | 0 | 2 | 2 | 14 | 58,33% |  | Medium risk |  |
| 18 | Hirata, 2014  <https://doi.org/10.1002/jbm.b.33258> | 2 | 1 | 2 | 1 | 2 | 0 | 0 | 2 | 0 | 0 | 1 | 2 | 13 | 54,17% |  | Medium risk |  |
| 19 | Hirokane E, 2021  <https://doi.org/10.2341/20-253-L> | 2 | 2 | 2 | 1 | 2 | 0 | 1 | 2 | 0 | 0 | 2 | 2 | 16 | 66,67% |  | Medium risk |  |
| 20 | Ilie N, 2013  <https://doi.org/10.2341/12-395-L> | 2 | 1 | 2 | 1 | 2 | 0 | 0 | 2 | 0 | 0 | 2 | 2 | 14 | 58,33% |  | Medium risk |  |
| 21 | Jung JH, 2017  <https://doi.org/10.2341/16-254-L> | 2 | 1 | 2 | 2 | 2 | 0 | 0 | 2 | 0 | 0 | 1 | 2 | 14 | 58,33% |  | Medium risk |  |
| 22 | Kamalak H, 2018  [10.4066/biomedicalresearch.29-18-314](https://doi.org/10.4066/biomedicalresearch.29-18-314) | 1 | 1 | 1 | 2 | 1 | 0 | 0 | 2 | 0 | 0 | 2 | 2 | 12 | 50% |  |  | High risk |
| 23 | Kim YJ, 2015  <http://dx.doi.org/10.1016/j.jdent.2015.02.002> | 2 | 1 | 2 | 1 | 2 | 0 | 0 | 2 | 0 | 0 | 2 | 2 | 14 | 58,33% |  | Medium risk |  |
| 24 | Kim YJ, 2016  <https://doi.org/10.2341/15-260-L> | 2 | 1 | 2 | 1 | 2 | 0 | 0 | 2 | 0 | 0 | 2 | 2 | 14 | 58,33% |  | Medium risk |  |
| 25 | Lassila L, 2019  <https://doi.org/10.1007/s10266-018-0405-y> | 2 | 1 | 2 | 1 | 2 | 0 | 0 | 2 | 0 | 0 | 2 | 2 | 14 | 58,33% |  | Medium risk |  |
| 26 | Lempel E, 2016  <https://doi.org/10.3390/ijms17050732> | 2 | 1 | 2 | 2 | 2 | 0 | 0 | 2 | 0 | 0 | 2 | 2 | 15 | 62,5% |  | Medium risk |  |
| 27 | Lempel E, 2019  <https://doi.org/10.1016/j.dental.2018.11.017> | 2 | 1 | 2 | 2 | 2 | 0 | 0 | 2 | 0 | 0 | 2 | 2 | 15 | 62,5% |  | Medium risk |  |
| 28 | Lampel E, 2021  <https://doi.org/10.1016/j.dental.2021.02.013> | 2 | 1 | 2 | 1 | 2 | 0 | 0 | 2 | 0 | 0 | 2 | 2 | 14 | 58,33% |  | Medium risk |  |
| 29 | Leprince J, 2014  <http://dx.doi.org/10.1016/j.jdent.2014.05.009> | 2 | 1 | 2 | 1 | 2 | 0 | 0 | 2 | 0 | 0 | 1 | 2 | 13 | 54,17% |  | Medium risk |  |
| 30 | Majidinia S, 2022  <https://doi.org/10.4103/denthyp.denthyp_41_19> | 2 | 1 | 2 | 1 | 2 | 0 | 0 | 2 | 0 | 0 | 2 | 2 | 14 | 58,33% |  | Medium risk |  |
| 31 | Marigo L, 2015  PMID: 26698240 | 2 | 1 | 2 | 1 | 2 | 0 | 0 | 2 | 0 | 0 | 2 | 2 | 14 | 58,33% |  | Medium risk |  |
| 32 | Marovic D, 2014  <https://doi.org/10.3109/00016357.2014.992810> | 2 | 1 | 2 | 2 | 2 | 0 | 0 | 2 | 0 | 0 | 2 | 2 | 15 | 62,5% |  | Medium risk |  |
| 33 | Monterubbianesi R, 2016  <https://doi.org/10.3389/fphys.2016.00652> | 2 | 1 | 2 | 1 | 2 | 0 | 0 | 2 | 0 | 0 | 2 | 2 | 14 | 58,33% |  | Medium risk |  |
| 34 | Nakano EL, 2000  <https://doi.org/10.2341/19-166-L> | 2 | 1 | 2 | 1 | 2 | 0 | 0 | 2 | 0 | 0 | 1 | 2 | 13 | 54,17% |  | Medium risk |  |
| 35 | Nitta K, 2017  <https://doi.org/10.4012/dmj.2016-394> | 2 | 1 | 2 | 1 | 2 | 0 | 0 | 2 | 0 | 0 | 2 | 2 | 14 | 58,33% |  | Medium risk |  |
| 36 | Oglakci B, 2020  <https://doi.org/10.1080/01694243.2020.1782038> | 2 | 2 | 2 | 1 | 2 | 0 | 0 | 2 | 0 | 0 | 2 | 2 | 15 | 62,50% |  | Medium risk |  |
| 37 | Oh Soram, 2022  <https://doi.org/10.1186/s40824-022-00267-5> | 2 | 1 | 2 | 1 | 2 | 0 | 0 | 2 | 0 | 0 | 2 | 2 | 14 | 58,33% |  | Medium risk |  |
| 38 | Öznurhan F, 2015  <https://doi.org/10.17796/1053-4628-39.3.241> | 2 | 1 | 2 | 1 | 2 | 0 | 0 | 2 | 0 | 0 | 1 | 2 | 13 | 54,17% |  | Medium risk |  |
| 39 | Papadogiannis D, 2015  <http://dx.doi.org/10.1016/j.dental.2015.09.022> | 2 | 1 | 2 | 1 | 2 | 0 | 0 | 2 | 0 | 0 | 1 | 2 | 13 | 54,17% |  | Medium risk |  |
| 40 | Par M, 2015  <https://doi.org/10.2341/14-091-L> | 2 | 1 | 2 | 1 | 2 | 0 | 0 | 2 | 0 | 0 | 2 | 2 | 14 | 58,33% |  | Medium risk |  |
| 41 | Prager M, 2018  <https://doi.org/10.4012/dmj.2017-136> | 2 | 1 | 2 | 1 | 2 | 0 | 0 | 2 | 0 | 0 | 2 | 2 | 14 | 58,33% |  | Medium risk |  |
| 42 | Rizzante, 2019  <http://dx.doi.org/10.1590/1678-7757-2018-0132> | 2 | 1 | 2 | 1 | 2 | 0 | 0 | 2 | 0 | 0 | 1 | 2 | 13 | 54,17% |  | Medium risk |  |
| 43 | Rizzante, 2019  <https://doi.org/10.4012/dmj.2018-063> | 2 | 1 | 2 | 1 | 2 | 0 | 0 | 2 | 0 | 0 | 1 | 2 | 13 | 54,17% |  | Medium risk |  |
| 44 | Sampaio C, 2016  <https://doi.org/10.2341/15-296-L> | 2 | 2 | 2 | 1 | 2 | 0 | 2 | 2 | 0 | 0 | 2 | 2 | 17 | 70,83% | Low risk |  |  |
| 45 | Sampaio C, 2019  <https://doi.org/10.1016/j.dental.2019.07.025> | 2 | 2 | 2 | 1 | 2 | 1 | 0 | 2 | 1 | 0 | 2 | 2 | 17 | 70,83% | Low risk |  |  |
| 46 | Sousa-Lima RX, 2017  <https://doi.org/10.2341/16-299-L> | 2 | 1 | 2 | 1 | 2 | 0 | 0 | 2 | 0 | 0 | 2 | 2 | 14 | 58,33% |  | Medium risk |  |
| 47 | Shimatani Y, 2020  <https://doi.org/10.2341/18-160-L> | 2 | 1 | 2 | 1 | 2 | 0 | 2 | 2 | 0 | 0 | 1 | 2 | 15 | 62,5% |  | Medium risk |  |
| 48 | Siagian J, 2020  <https://doi.org/10.5005/jp-journals-10024-2848> | 2 | 1 | 2 | 1 | 2 | 0 | 0 | 2 | 0 | 0 | 1 | 2 | 13 | 54,17% |  | Medium risk |  |
| 49 | Theobaldo J, 2017  <https://doi.org/10.2147/CCIDE.S130803> | 2 | 1 | 2 | 2 | 2 | 1 | 0 | 2 | 0 | 0 | 1 | 2 | 15 | 62,5% |  | Medium risk |  |
| 50 | Tsujimoto A, 2021  <https://doi.org/10.3390/polym13162613> | 1 | 1 | 2 | 1 | 2 | 0 | 0 | 2 | 0 | 0 | 2 | 2 | 13 | 54,17% |  | Medium risk |  |
| 51 | Velo M, 2019  <http://dx.doi.org/10.1590/0103-6440201902571> | 2 | 2 | 2 | 1 | 2 | 0 | 0 | 2 | 0 | 0 | 2 | 2 | 15 | 62,5% |  | Medium risk |  |
| 52 | Yu P, 2017  <https://doi.org/10.2341/16-027-L> | 1 | 1 | 2 | 1 | 2 | 0 | 0 | 2 | 0 | 0 | 2 | 2 | 13 | 54,17% |  | Medium risk |  |
| 53 | Zorzin J, 2015  <http://dx.doi.org/10.1016/j.dental.2014.12.010> | 2 | 1 | 2 | 1 | 2 | 0 | 0 | 2 | 0 | 0 | 2 | 2 | 14 | 58,33% |  | Medium risk |  |
|  |  |  |  |  |  |  |  |  |  |  |  |  |  |  |  |  |  |  |

Criteria of QUIN Tool. C1: Clearly stated aims/objectives; C2: Detailed explanation of sample size calculation; C3: Detailed explanation of sampling technique; C4: Detail of comparison group; C5: Detailed explanation of methodology; C6: Operator details; C7: Randomization; C8: Method of measurement of outcome; C9: Outcome assessor details; C10: Blinding; C11: Statistical analysis and C12: Presentation of results. TS: Total score, corresponds to the sum of the evaluation of each of the criteria (2 points when adequately specified, 1 point when inadequately specified, and 0 point when not specified. A criterion can be excluded from the calculation when the criteria is not applicable). %RoB: is the result, in percentage of the risk of bias when applying the formula “Final Score = (Total score x 100) / (2 x number of criteria applicable. The obtained score can be graded as “high risk” when %RoB is <50%, “medium risk” when %RoB is >50<70%, and “low risk” when %RoB is >70%.
